# Lymphocyte-to-monocyte ratio after primary surgery is an independent prognostic factor for patients with epithelial ovarian cancer: A propensity score matching analysis

**DOI:** 10.3389/fonc.2023.1139929

**Published:** 2023-03-22

**Authors:** Qian Hu, Guihua Shen, Ye Li, Ya Xie, Xiao Ma, Lijuan Jiang, Qiubo Lv

**Affiliations:** ^1^ Department of Obstetrics and Gynecology, Beijing Hospital, National Center of Gerontology, Institute of Geriatric Medicine, Chinese Academy of Medical Sciences, Beijing, China; ^2^ Gynecology Department, The First Affiliated Hospital of Zhengzhou University, Zhengzhou, China; ^3^ Department of Obstetrics and Gynecology, Beijing Pinggu Hospital, Beijing, China; ^4^ Department of Obstetrics and Gynecology, Shunyi Maternal and Children’s Hospital of Beijing Children’s Hospital, Beijing, China

**Keywords:** lymphocyte-to-monocyte ratio, epithelial ovarian cancer, propensity score matching, adjuvant chemotherapy, overall survival

## Abstract

**Background:**

The aim of this study was to elucidate the prognostic value of preoperative lymphocyte-to-monocyte ratio (LMR) after primary surgery in epithelial ovarian cancer (EOC) patients using a propensity score matching (PSM) analysis.

**Methods:**

We retrospectively reviewed consecutive EOC patients who underwent primary surgery between January 2008 and December 2019. Patients were divided into two groups according to the optimal cutoff value of preoperative LMR. PSM (1:1) was conducted to eliminate confounding factors. A Cox proportional hazards model and the Kaplan–Meier estimator were employed to investigate the potential prognostic factors.

**Results:**

A total of 368 EOC patients were included in this study. The optimal cutoff value of LMR was identified as 4.65. Low preoperative LMR was significantly correlated with low albumin, high CA125 level, more blood loss, a high likelihood of ascites, advanced FIGO stage, and poor differentiation (all *p* < 0.05). After matching, Kaplan–Meier curves showed that the group with LMR < 4.65 experienced significantly shorter OS (*p* = 0.015). Multivariate Cox analysis revealed that low LMR (HR = 1.49, *p* = 0.041), advanced FIGO stage (HR = 5.25, *p* < 0.001), and undefined residual disease (HR = 3.77, *p* = 0.002) were independent factors in predicting poor OS. A forest plot revealed that LMR had better prognostic value in younger EOC patients, patients with BMI ≥ 25 kg/m^2^ and albumin ≥ 35 g/L, CA125 ≥ 35 U/L, patients who had undergone optimal surgery, and those who had completed chemotherapy. Additionally, low-LMR patients who had undergone incomplete chemotherapy had a shorter median OS compared with those who completed chemotherapy treatment (48.5 *vs*. 105.9 months, *p* = 0.026).

**Conclusions:**

LMR could be used as an independent prognostic factor for EOC patients after primary surgery; a noticeable negative effect of LMR was observed among EOC patients with age < 65, good preoperative nutritional status, and more aggressive tumor biology, and among those who underwent optimal surgery. Completing adjuvant chemotherapy is essential to improve survival outcomes among EOC patients with LMR < 4.65 after surgery.

## Introduction

Ovarian cancer is the fifth most common malignancy in women and remains the leading cause of death among gynecologic malignancies (1). Most patients present at an advanced stage, and 85% of them will experience recurrence within 2 years after receiving primary treatment (2). Despite great advances in therapeutic strategy, the 5-year survival rate has changed little over the past decades, remaining at only 30% (3). Epithelial ovarian cancer (EOC) accounts for more than 90% of ovarian cancer and identification of prognostic factors for EOC is crucial for appropriate patient management.

Classic prognostic factors for EOC, such as residual tumor size, histopathological results, platinum sensitivity, and molecular features, are only available in the postoperative setting (4–6). Many immunological and nutritional markers have been reported to be prognostic factors for ovarian cancer (7). Among these, lymphocyte-to-monocyte ratio (LMR), which is calculated from white blood cell differential counts, can be easily obtained in preoperative patients. Previous studies have reported on the predictive potential of LMR as a prognostic factor in various types of malignancies, including ovarian cancer (8–11). However, the results of some studies have been inconsistent (12, 13). Furthermore, it is unclear whether variables such as surgical effect, chemotherapy, and disease stage influence the ability of LMR to predict prognosis of patients with resectable ovarian cancer. To address these issues and to increase the strength of the evidence, the present study aimed to investigate the correlation of LMR in patients with EOC who underwent primary surgery with long-term oncologic outcomes using a propensity score matching (PSM) analysis.

## Materials and methods

### Patient selection

The medical records of 399 patients who underwent primary surgery for ovarian cancer at Beijing Hospital between January 2008 and December 2019 were retrospectively reviewed. This study was approved by the Institutional Review Board of Beijing Hospital (IRB No: 2022BJYYEC-227-02). Before conducting the data analysis, we reclassified the patients according to the FIGO staging guidelines (2014) and the WHO classification (14, 15). Of the full set of patients, 31 were excluded from this study due to comorbidity of other malignancies or incomplete clinicopathological or follow-up data (*n* = 31). The remaining 368 patients were enrolled in the study. Patient demographics, Eastern Cooperative Oncology Group performance status (ECOG PS), blood sample results, information on residual disease, and other clinical–pathological parameters were extracted from electronic medical records. All biochemical tests were performed within 1 week prior to surgery for resection of the primary tumor (initial treatment). Each patient underwent enhanced computed tomography (CT), magnetic resonance imaging (MRI), or positron emission tomography/computed tomography (PET/CT) before surgery.

### Lymphocyte count/absolute monocyte count

**Figure 1:**
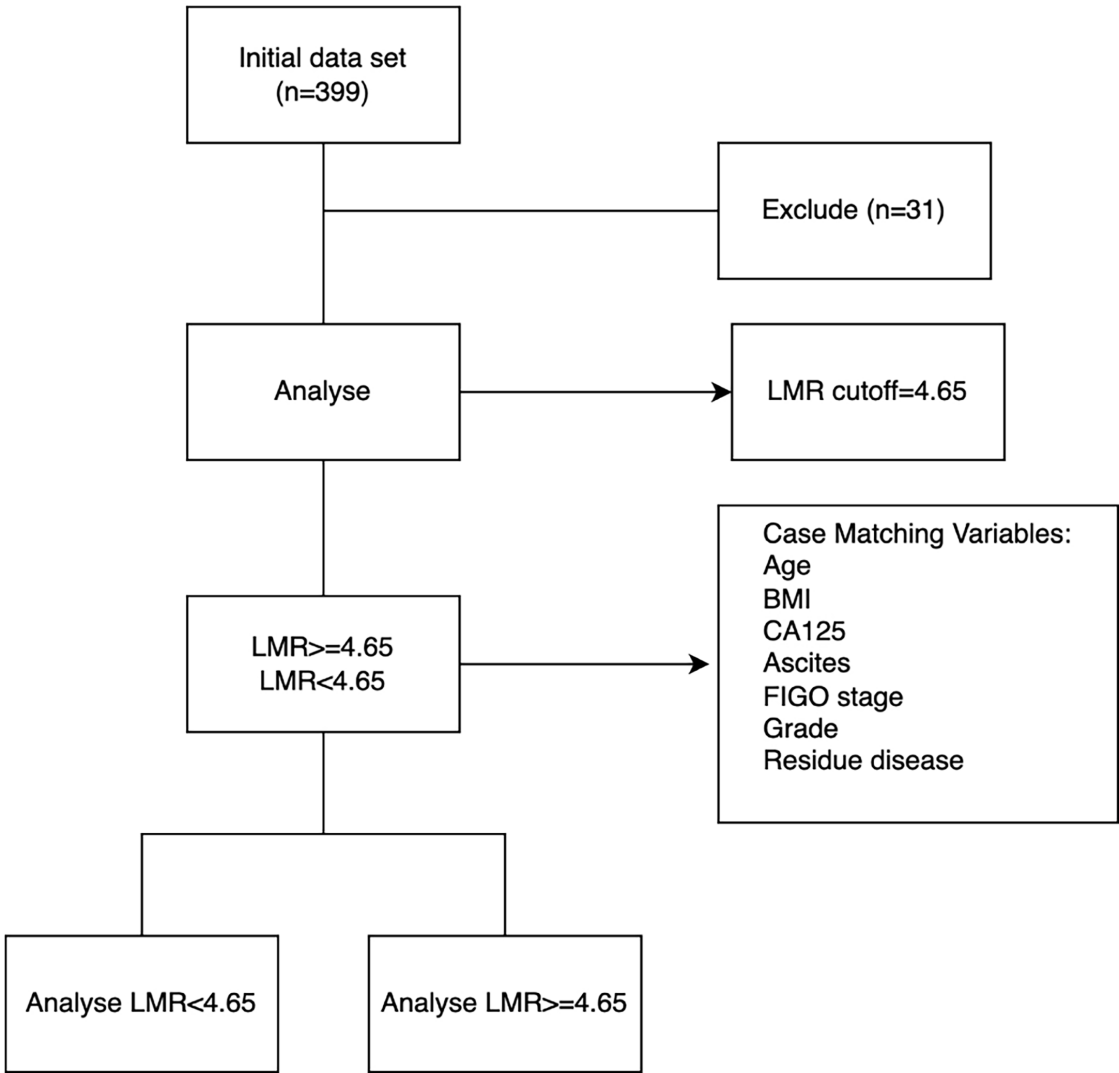
Flowchart of the study population.

LMR is derived by dividing absolute lymphocyte count by absolute monocyte count. The cutoff value for mortality was determined using receiver operating characteristic (ROC) analysis. All patients were allocated to either a the low-LMR group or a high-LMR group for subsequent analyses. A flow diagram illustrating subject screening and grouping is provided in **Figure 1**.

### Surgical and adjuvant chemotherapy

All patients underwent primary curative surgery (staging or debulking). Residual disease was defined as all macroscopic visible residual tumor in the abdominopelvic cavity and categorized as (1): optimal (R0 = no visible tumor, R1 = residual tumor < 1 cm) (2), suboptimal (residual tumor ≥ 1 cm), or (3) no data (data not available) (16).

Postoperative systemic adjuvant chemotherapy was generally performed according to NCCN guidelines in ovarian cancer; most of the patients received four to eight cycles of adjuvant platinum-based chemotherapy. The most common chemotherapy regimens used were paclitaxel/carboplatin and paclitaxel/nedaplatin. Completion of adjuvant chemotherapy was defined as receiving ≥6 cycles of postoperative chemotherapy.

### Follow-up

For patients who were followed up at our institute, follow-up included physical examination, serum CA125 testing, and imaging evaluation every 3 months during the first 2 years; the interval was extended to 6 months for the next 3 years, and then once a year thereafter. Recurrence was defined as unequivocal radiologic evidence of progression of residual tumor or emerging new tumor lesion, with or without elevated CA125. The site and date of the first recurrence were recorded.

### Statistical analysis

The primary outcome was 5-year overall survival (OS). OS was defined as the interval between the date of operation and the date of either death or the end of the observation period. Patients alive at the end of follow-up were recorded as censored. Survival characteristics were assessed using the Kaplan–Meier method, and the groups were compared using the log-rank test. The Cox proportional hazards model was used to evaluate the hazard ratios for death. Variables that were statistically significant (*p* < 0.05) in univariate Cox regression analyses were incorporated into a multivariate analysis to identify independent prognostic factors for survival. Forest plots were constructed to show the outcome of subgroup analysis. Propensity score matching analyses were performed to balance the significant variables used in the analyses between two groups allocated by LMR. Propensity scores were estimated using a logistic regression model according to age, preoperative body mass index (BMI), ascites, CA125 level, FIGO stage, grade, and residual disease; 1:1 matching without replacement was performed using the nearest-neighbor matching method with 0.6 caliper width, and the resulting score-matched pairs were used in subsequent analyses. All statistical analyses were performed using R software, version 4.2.1 (https://www.r-project.org/). A *p*-value < 0.05 was considered to represent statistical significance.

## Results

### Patient characteristics

A total of 368 patients diagnosed with EOC who accepted primary surgery were included in the full cohort. The patients were followed up for a median period of 82.8 months (range: 72.9–95.0 months).

According to ROC curve analyses, the optimum cutoff for the LMR value to predict 5-year OS in EOC, yielding maximum sensitivity and specificity, was 4.65 (AUC = 0.635, 95% CI = 0.343–0.698). Using this cutoff, we classified all 368 patients into groups with LMR < 4.65 (*n* = 245) or LMR ≥ 4.65 (*n* = 123). Before propensity score matching, low LMR was associated with higher rate of presence of ascites, more advanced tumor stage, and higher CA125 level (**Table 1**). Propensity score matching was performed to minimize selection bias, and the baseline characteristics of the two groups (*n* = 111 each) became well-balanced, as demonstrated by a covariate balance plot and a histogram (**Figure S1**). The median follow-up periods were 75.2 months (range: 66.3–85.9 months) and 102.4 months (range: 81.0–118.5 months) for the LMR < 4.65 and LMR ≥ 4.65 groups, respectively.

**Table 1:** Demographics and pathological characteristics of patients in each group before and after propensity score matching.

Characteristic	Before PSM			After PSM		
	LMR < 4.65 (*n* = 245)	LMR ≥ 4.65 (*n* = 123)	*p*-value	LMR < 4.65 (*n* = 111)	LMR ≥ 4.65 (*n* = 111)	*p*-value
Age (years), mean ± SD	56.9 (11.2)	56.3 (10.1)	0.568	56.6 (11.4)	56.8 (10.2)	0.921
BMI (kg/cm^2^), mean ± SD	24.9 (15.5)	24.1 (3.5)	0.566	23.7 (3.7)	23.9 (3.5)	0.606
ECOG						
0	110 (44.9)	57 (46.3)	0.941	49 (44.1)	51 (45.9)	0.685
1	113 (46.1)	54 (43.9)		54 (48.6)	49 (44.1)	
2	19 (7.8)	11 (8.9)		8 (7.2)	11 (9.9)	
3	3 (1.2)	1 (0.8)		0 (0.0)	0 (0.0)	
Ascites						
No	117 (47.8)	84 (68.3)	<0.001	80 (72.1)	77 (69.4)	0.768
Yes	128 (52.2)	39 (31.7)		31 (27.9)	34 (30.6)	
Albumin (g/dl), mean ± SD	37.8 (5.1)	40.6 (4.2)	<0.001	38.3 (5.6)	40.5 (4.4)	0.001
Preoperative CA-125 (U/ml)	563.8 (195.5, 1,801.6)	149.2 (37.3, 654.5)	<0.001	339.20 (76.0, 606.0)	149.20 (37.3, 647.4)	0.048
Operative time (min), median (IQR)	180.0 (140.0, 225.0)	185.0 (140.0, 210.0)	0.926	180.0 (137.5, 235.0)	185.0 (142.5, 210.0)	0.801
Blood loss (ml), median (IQR)	800.0 (500.00, 1,000.0)	600.0 (400.0, 800.0)	0.001	600.0 (350.0, 900.0)	600.0 (400.0, 800.0)	0.668
Maximum tumor size (cm), median (IQR)	6.00 (4.0, 9.5)	7.0 (5.0, 10.0)	0.062	6.0 (4.0, 10.0)	7.0 (5.0, 10.0)	0.361
Residual disease, *n* (%)						
Optimal	195 (79.6)	110 (89.4)	0.042	97 (87.4)	98 (88.3)	0.974
Suboptimal	44 (18.0)	10 (8.1)		11 (9.9)	10 (9.0)	
No data	6 (2.4)	3 (2.4)		3 (2.7)	3 (2.7)	
FIGO stage, *n* (%)						
I	35 (14.3)	44 (35.8)	<0.001	35 (31.5)	36 (32.4)	0.950
II	22 (9.0)	28 (22.8)		22(19.8)	24 (21.6)	
III	150 (61.2)	44 (35.8)		48 (43.2)	44 (39.6)	
IV	38 (15.2)	7 (5.7)		6 (5.4)	7 (6.3)	
Grade, *n* (%)						
G1	10 (4.1)	5 (4.1)	0.010	4 (3.6)	2 (1.8)	0.102
G2	64 (26.1)	51 (41.5)		36 (32.4)	51 (45.9)	
G3	171 (69.8)	67 (54.5)		71 (64.0)	58 (52.3)	
Histology, *n* (%)						
Serous	195 (79.6)	84 (68.3)	0.114	74 (66.7)	77 (69.4)	0.607
Mucinous	8 (3.3)	9 (7.3)		6 (5.4)	9 (8.1)	
Endometrioid	11 (4.5)	10 (8.1)		10 (9)	10 (9)	
Clear cell	20 (8.2)	11 (8.9)		16 (14.4)	9(8.1)	
Other	11(4.5)	9 (7.3)		5 (4.5)	6 (5.4)	
Adjuvant chemotherapy, *n* (%)						
Absent	24 (9.8)	10 (8.1)	0.156	15 (13.5)	6 (5.4)	0.058
Incomplete	57 (23.3)	19 (15.4)		22 (19.8)	17 (15.3)	
Complete	164 (66.9)	94 (76.4)		74 (66.7)	88 (79.3)	

### Prognostic significance of LMR in EOC

**Figure 2:**
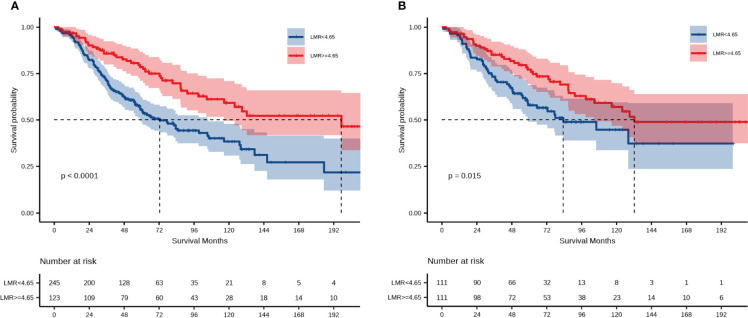
Prognostic significance of LMR for OS. **(A)** Overall survival among the entire cohort; **(B)** overall survival among the PSM cohort.

Kaplan–Meier analysis over the entire cohort demonstrated that the low-LMR group experienced significantly shorter OS (*p* < 0.0001, **Figure 2A**). The median OS among subjects with LMR < 4.65 and those with LMR ≥ 4.65 was 72.5 months and 197.4 months, respectively. Univariate Cox regression analysis indicated that LMR, age, ascites, CA125 level, blood loss, residual disease, and FIGO stage were independent prognostic factors. These factors were subsequently incorporated into a multivariate Cox regression analysis. As shown in **Table 2**, LMR was identified as a candidate risk factor (HR = 1.49, 95% CI: 1.02–2.18, *p* = 0.041). In the PSM cohort, the survival analysis further confirmed that patients with LMR < 4.65 had shorter OS compared with patients with LMR ≥ 4.65 (*p* = 0.015, **Figure 2B**). The median OS of the low- and high-LMR groups was 83.3 months and 132.2 months, respectively. Moreover, the multivariate Cox analysis demonstrated that residual disease (no data: HR = 3.77, 95% CI: 1.65–8.63; *p* = 0.002) and FIGO stage (II: HR = 3.04, 95% CI: 1.40–6.60, *p* = 0.005; III/IV: HR = 5.25, 95% CI: 2.62–10.50, *p* < 0.001) were independent prognostic factors.

**Table 2:** Univariate and multivariate Cox regression analysis of clinical parameters for overall survival.

Variables	Univariate			Multivariate		
	HR	95% CI	*p*-value	HR	95% CI	*p*-value
LMR (ref ≥ 4.65)	2.04	1.43–2.91	<0.001	1.49	1.02–2.18	0.041
Age, years (ref ≤ 65)	1.47	1.04–2.07	0.027	1.27	0.89–1.8	0.188
BMI, kg/m^2^ (ref ≤ 25)	1.13	0.83–1.54	0.447			
ECOG (ref ≤ 2)	0.99	0.24–3.99	0.984			
Ascites (ref = no)	1.76	1.29–2.39	<0.001	1.04	0.74–1.46	0.814
Albumin, g/dl (ref ≤ 35)	0.73	0.51–1.04	0.081			
CA-125, U/ml (ref ≤ 35)	2.84	1.57–5.11	<0.001	1.14	0.59–2.20	0.687
Tumor size, cm (ref ≤ 6)	0.81	0.59–1.1	0.174			
Blood loss, ml (ref ≤ 600)	1.41	1.03–1.92	0.029	0.93	0.67–1.29	0.668
Residual disease (ref = suboptimal)						
Optimal	0.73	0.47–1.13	0.163	1.13	0.72–1.77	0.595
No data	2.58	1.16–5.75	0.020	3.77	1.65–8.63	0.002
FIGO stage (ref = I)						
II	2.92	1.39–6.13	0.005	3.04	1.4–6.6	0.005
III and IV	6.18	3.34–11.43	<0.001	5.25	2.62–10.5	<0.001
Grade (ref = G1)						
G2	0.97	0.42–2.27	0.945			
G3	1.40	0.61–3.21	0.422			
Adjuvant chemotherapy (ref = absent)						
Incomplete	1.25	0.72–2.17	0.430			
Complete	0.66	0.40–1.07	0.091			

### Subgroup analysis

**Figure 3:**
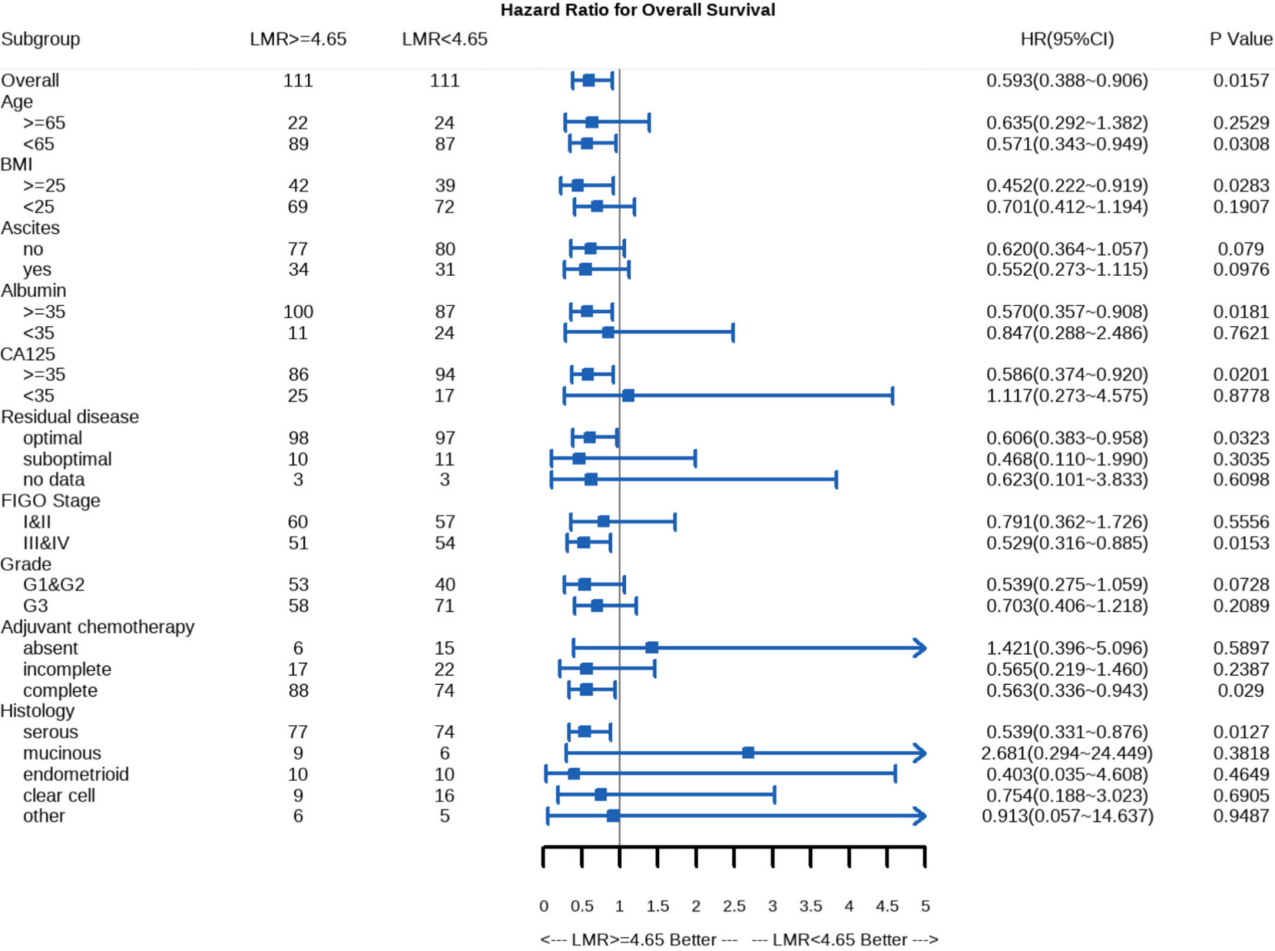
Significance of the association of LMR with overall survival in the PSM cohort.

Further subgroup analyses were performed to explore whether LMR remained as a prognostic factor in certain subgroups. Forest plots revealed that LMR < 4.65 may be associated with a poorer prognosis in younger patients (HR = 0.571, 95% CI: 0.343–0.949, *p* = 0.031), patients with a BMI of more than 25 kg/cm^2^ (HR = 0.452, 95% CI: 0.222–0.919, *p* = 0.028), patients with an albumin level of more than 35 g/L (HR = 0.570, 95% CI: 0.357–0.908, *p* = 0.018), patients with a CA125 level higher than 35 U/ml (HR = 0.586, 95% CI: 0.374–0.920, *p* = 0.020), those with stage III/IV disease (HR = 0.529, 95% CI: 0.316–0.885, *p* = 0.015), those with serous adenocarcinoma (HR = 0.539, 95% CI: 0.331–0.876, *p* = 0.0127), patients who have undergone optimal surgery (HR = 0.606, 95% CI: 0.383–0.958, *p* = 0.032), and those who completed postoperative adjuvant chemotherapy (HR = 0.563, 95% CI: 0.336–0.943, *p* = 0.029) (**Figure 3**).

**Figure 4:**
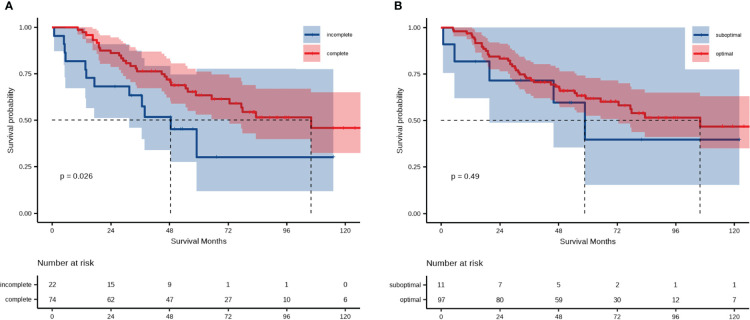
Analysis of overall survival of patient groups categorized according to LMR and **(A)** administration of postoperative adjuvant chemotherapy or **(B)** residual disease.

Following this, we focused on residual disease status and on administration of adjuvant chemotherapy in patients with LMR < 4.65. The 5-year OS rates were 30.0% and 63.0% for patients in the LMR < 4.65 group receiving complete and incomplete adjuvant chemotherapy, respectively (**Figure 4A**). There were no statistically significant differences in OS within the LMR < 4.65 group, regardless of residual disease status (**Figure 4B**).

## Discussion

The host immune system has an important impact on cancer development and progression (17, 18). Lymphocytes play a key protective role in the adaptive immune system, and a decreased lymphocyte count has been found to be broadly associated with poor prognosis in many malignancies (19, 20). There is increasing evidence that lymphocytes exert a specific cytotoxic antitumor effect and promote the antitumor response by differentiating into tumor-infiltrating lymphocytes (21, 22). Intratumoral CD8+ T lymphocytes have been linked to improved OS and identified as an independent prognostic factor (23). In contrast, monocytes can contribute to promoting tumorigenesis and suppressing the immune response in cancer by differentiating to tumor-associated macrophages (TAMs) and dendritic cells (18, 21). TAMs have also been identified as critical in the biology of cervical (24), endometrial (25), and ovarian cancer (26). Tumor-induced monocytes play an essential role in tumor progression by activating the epithelial–mesenchymal transition process (27). Accordingly, peripheral immune cell counts reflect direct qualitative and quantitative interactions at the tumor level, and the imbalance in circulating lymphocyte and monocytes indexes variation in the general immune condition of the host (28).

Previous studies have demonstrated the predictive potential of LMR in evaluating the prognosis of patients with various types of malignancies, including resectable ovarian cancer (8, 9, 27–29). In 2016, Eo et al. first reported a correlation between low preoperative LMR and poor prognosis in ovarian cancer (9). However, to the best of our knowledge, few studies have analyzed long-term prognosis in a large cohort of patients with advanced ovarian cancer using propensity scores to minimize selection bias. In the present study, the cohort consisted of 368 patients with ovarian cancer, and after case matching according to propensity scores, significant differences were observed between the low-LMR and high-LMR groups in terms of OS. Cutoff values for LMR range from 1.85 to 5.2 (8–10, 30, 31). In accordance with these findings, LMR was found to predict OS prognosis with a cutoff value of 4.65 in the present study.

A higher proportion of patients with advanced-stage disease, poor differentiation, and high CA125 was observed in the low-LMR group, which was consistent with previous reports. Wang et al. demonstrated that low preoperative LMR is associated with higher pathological grade, more advanced FIGO stage, lymph node metastasis, and inferior OS in patients with prostate cancer (11). This suggests that lymphocytes are important to eradicate residual tumor cells and related micrometastases, and tumor-infiltrating lymphocytes provide a defensive barrier against cancer dissemination (32, 33). In contrast, the infiltration of monocytes into tumor tissue has been shown to accelerate tumor cell growth and invasion in lymphoma (34). LMR may reflect the imbalance of these two types of cells during cancer development, and thus can be considered to be a surrogate biomarker for OS.

Subgroup analyses demonstrated that LMR remained a prognostic factor in EOC patients with serous adenocarcinoma, good nutritional status (high albumin), and relatively high tumor burden (stage III/IV and high CA125). These results indicated that the prognostic effect of LMR was still apparent among patients with aggressive cancer, which would predominantly worsen the survival outcome (4). Therefore, LMR can serve as a risk stratification factor for advanced high-grade serous adenocarcinoma patients, contributing to individualized diagnosis and treatment. Interestingly, our study found that the outcome of survival favored the high-LMR group in the subgroup of patients aged < 65. Despite the findings not being significant in the elderly group (age ≥ 65), we speculated that biological aging is linked to a decline in immune responses, which led to the absence of any clear association in elderly patients (35).

Most EOC patients need postoperative adjuvant chemotherapy after surgery. The prognostic impact of adjuvant chemotherapy in EOC patients following radical resection has been well recognized. However, there is still controversy regarding whether the number of chemotherapy cycles is a prognostic factor for EOC patients, and whether patients with early-stage EOC would benefit from adjuvant chemotherapy (36–38). Our study showed that low-LMR patients who had not completed chemotherapy had worse OS after matching (median OS, incomplete chemotherapy *vs*. complete chemotherapy, 48.5 *vs*. 105.9 months), indicating that LMR may furnish crucial information on the individual value of chemotherapy in EOC patients. Previous evidence has also indicated residual disease to be an independent prognostic marker for OS in EOC patients (4). Although no significant differences were apparent between the optimal and suboptimal groups in patients with LMR < 4.65, there seemed to be a downward trend in OS for patients with suboptimal surgery. It has been previously indicated that size of residual disease (no macroscopic residual disease vs. 0.1–1 cm vs. >1 cm) after primary surgery is a prognostic factor for OS and progression-free survival in women with advanced ovarian cancer (39). It is possible that the combination of R0 and R1 patients is one reason for the lack of association in the present study. An alternative explanation is that the limited number of patients undergoing suboptimal cytoreductive surgery after PSM might have exerted an influence on this result.

This study has several limitations. First, this was a single-center retrospective study. To minimize selection bias, we conducted a propensity score matching analysis; however, 1:1 matching caused a substantial loss of data, so the results may not accurately reflect the target population. Future studies with multicenter, prospective, and large-scale designs are warranted to adequately generalize our findings. Additionally, our study was underpowered to detect a difference between groups with different degrees of residual disease after surgery; further research is required to confirm this comparison. Despite this, a downward trend in OS for patients with suboptimal surgery was observed. Finally, molecular assessment, such as BRCA mutation, was not included in this study. Since most patients included were treated before routine detection of genetic testing was performed in EOC patients in China, the adoption rate was insufficient, but this did not alter the interpretation of the results.

In summary, LMR could be used as an independent prognostic factor for EOC patients after primary surgery. A noticeable negative effect of LMR was observed among EOC patients with age < 65 years, better preoperative nutritional status, and more aggressive tumor biology. Complete adjuvant chemotherapy is essential to improve survival outcomes among EOC patients with LMR < 4.65 after surgery.

## Data availability statement

The datasets generated during and/or analyzed during the current study are available from the corresponding author on reasonable request. Requests to access the datasets should be directed to flushaa@163.com.

## Author contributions

Conceptualization: QH. Data curation: XM and LJ. Formal analysis: GS and YL. Investigation: QH, GS, YL, and YX. Methodology: QH and GS. Project administration: QL. Supervision: QL. Writing – original draft: QH. Writing – review and editing: GS, YL, YX, and QL. All authors contributed to the article and approved the submitted version.
