# Supplementary figures and images for: Lymphocyte-to-monocyte ratio after primary surgery is an independent prognostic factor for patients with epithelial ovarian cancer: A propensity score matching analysis
Source: Front Oncol. 2023 Mar 22;13:1139929. doi: 10.3389/fonc.2023.1139929 (PMC10075326; doi:10.3389/fonc.2023.1139929)

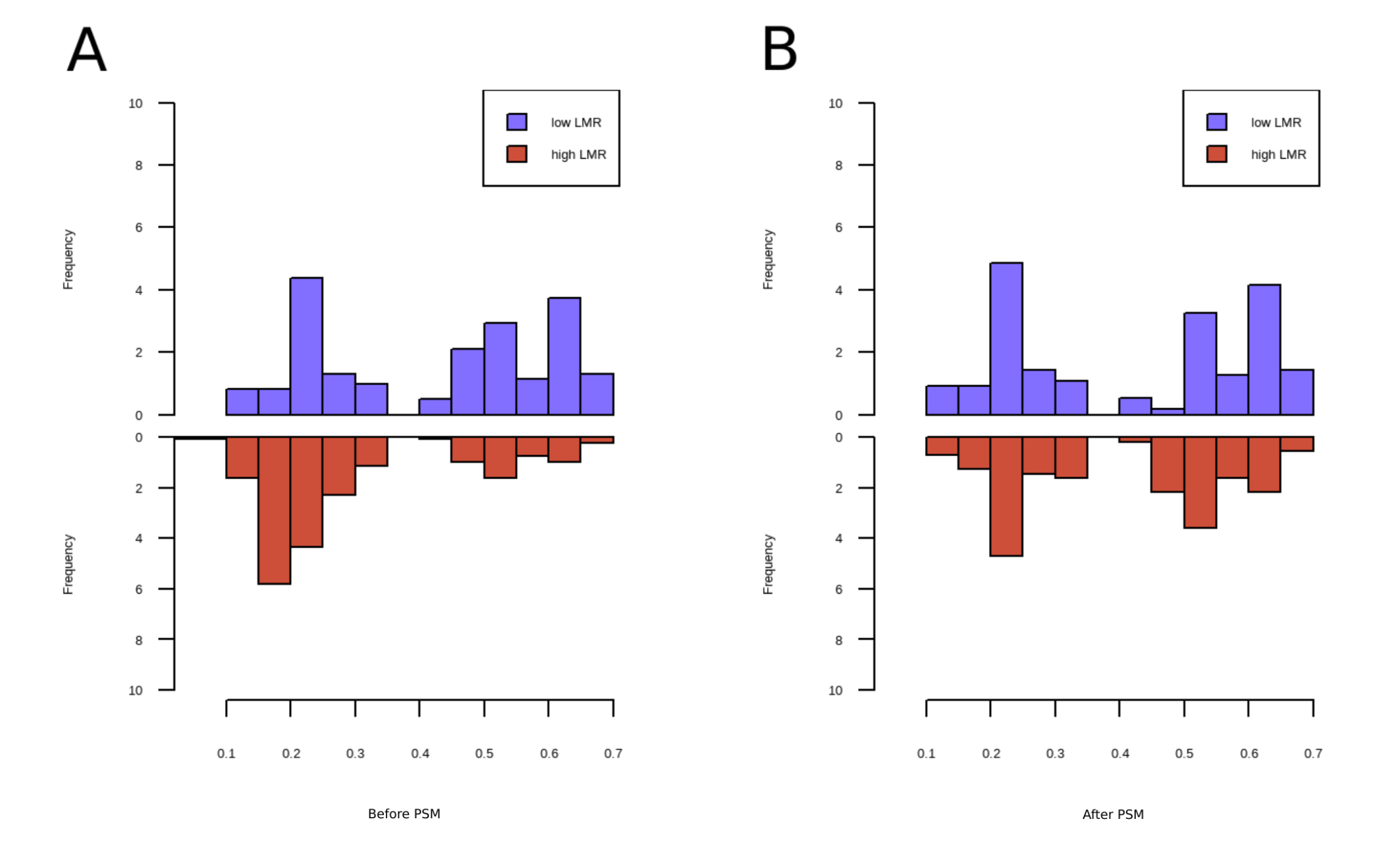

Supplement: Supplementary file 1 [file Image_1.jpeg]
